# Biochemical Characterization and Functional Analysis of Heat Stable High Potential Protease of *Bacillus amyloliquefaciens* Strain HM48 from Soils of Dachigam National Park in Kashmir Himalaya

**DOI:** 10.3390/biom11010117

**Published:** 2021-01-18

**Authors:** Hina Mushtaq, Arshid Jehangir, Shabir Ahmad Ganai, Saleem Farooq, Bashir Ahmad Ganai, Ruqeya Nazir

**Affiliations:** 1 Department of Environmental Science, University of Kashmir, Srinagar, Jammu & Kashmir 190006, India; heyna.6@gmail.com (H.M.); rathersaleem111@gmail.com (S.F.); 2 Division of Basic Sciences and Humanities, Faculty of Agriculture, SKUAST-Kashmir, Sopore 193201, Jammu & Kashmir, India; shabir.muntazir82@gmail.com; 3 Centre of Research for Development, University of Kashmir, Srinagar, Jammu & Kashmir 190006, India; ruqeya.ku@gmail.com

**Keywords:** Kashmir Himalaya, soil bacteria, 16S rRNA, K_M_, V_max_, alkaline protease, protein modeling, protein–protein docking

## Abstract

A novel temperature stable alkaline protease yielding bacteria was isolated from the soils of Dachigam National Park, which is known to be inhabited by a wide variety of endemic plant and animal species of Western Himalaya. This high-potential protease producing isolate was characterized and identified as *Bacillus amyloliquefaciens* strain HM48 by morphological, Gram’s staining and biochemical techniques followed by molecular characterization using 16S rRNA approach. The extracellular protease of *B. amyloliquefaciens* HM48 was purified by precipitating with ammonium sulfate (80%), followed by dialysis and Gel filtration chromatography increasing its purity by 5.8-fold. The SDS–PAGE analysis of the purified enzyme confirmed a molecular weight of about ≈25 kDa. The enzyme displayed exceptional activity in a broad temperature range (10–90 °C) at pH 8.0, retaining its maximum at 70 °C, being the highest reported for this proteolytic *Bacillus* sp., with K_M_ and V_max_ of 11.71 mg/mL and 357.14 µmol/mL/min, respectively. The enzyme exhibited remarkable activity and stability against various metal ions, surfactants, oxidizing agent (H_2_O_2_), organic solvents and displayed outstanding compatibility with widely used detergents. This protease showed effective wash performance by exemplifying complete blood and egg-yolk stains removal at 70 °C and efficiently disintegrated chicken feathers making it of vital importance for laundry purpose and waste management. For functional analysis, protease gene amplification of strain HM48 yielded a nucleotide sequence of about 700 bp, which, when checked against the available sequences in NCBI, displayed similarity with subtilisin-like serine protease of *B. amyloliquefaciens*. The structure of this protease and its highest-priority substrate β-casein was generated through protein modeling. These protein models were validated through futuristic algorithms following which protein–protein (protease from HM48 and β-casein) docking was performed. The interaction profile of these proteins in the docked state with each other was also generated, shedding light on their finer details. Such attributes make this thermally stable protease novel and suitable for high-temperature industrial and environmental applications.

## 1. Introduction

Dachigam National Park (henceforth, DNP) situated in the Zabarwan range of Himalayas lies to the north-east part of Srinagar, the state capital of Jammu and Kashmir, India. The park is ecologically significant as it is known to harbor a unique variety of plants and animals, including the critically endangered Kashmir red stag, locally known as “Hangul”, endangered Himalayan black bear, Himalayan brown bear, Himalayan gray langur, musk deer and snow leopard. The national park has been extensively studied for its phytodiversity and soil physicochemical characteristics [1], as well as its endangered animals [2,3,4], but is poorly understood as far as its soil bacterial studies are concerned. The molecular level study on bacterial diversity in forest ecosystems of Kashmir revealed many novel bacterial lineages [5]. However, the molecular characterization of soil bacterial populations from lower DNP, particularly for their enzyme bioprospection, has not been investigated yet. Although the great Himalayan mountain range is expected to contain a wide variety of proteolytic bacteria that could remain active over a wide range of temperatures, limited studies mainly on the cold-active proteases from psychrophilic bacteria [6,7] have been conducted. Thus, there is a need to prospect heat-stable proteases for high-temperature operations where heat acts as a limiting factor for carrying out the reactions efficiently.

Protease enzymes being among the three major industrial enzyme groups, make up for around 65% of the gross enzyme trading globally [8] and are envisaged in the development of several eco-friendly bio-remedial technologies [9,10]. Although a wide variety of soil microbes produce alkaline proteases [11,12], one of the main drawbacks of these proteases is their unstable nature in alkaline pH at higher temperatures [13] as these conditions are deleterious for most of the enzymes. Thus, there is a need to prospect new heat-stable alkaline proteases having novel properties owing to growing enzyme demand with better stability [14].

Proteases also called proteinases and peptidases [15], are a class of enzymes catalyzing the total protein hydrolysis into peptides and amino acids by cleaving their peptide bonds [16]. Gupta et al. [17] recognized protease as the most frequently used biocatalysts commercially, that occupy a vital place on account of their utilization in numerous physiologic and mercantile operations [18] like pharmaceuticals, fine chemical production, bioethanol production, and detergent industries [19]. Microbial proteases in comparison to the plant and animal sources of proteases [17] are more advantageous by virtue of their fast growth, vast diversity, ease of cultivation and genetic manipulations [20]. Approximately two-thirds of the marketable proteases are obtained from several species of bacteria, fungi, and yeasts, of which bacteria-derived proteolytic enzymes, particularly from *Bacillus* species, are industrially significant, accounting for about 35% of microbial enzymes [10]. However, for successful industrial application, proteases must be stable and function at elevated temperatures as well as pH [21].

Of various protease types-acidic, neutral and alkaline [17], proteases belonging to the alkaline type being active and stable at elevated pH are the most frequently used commercial enzymes [22]. Alkaline proteases, EC.3.4.21-24;99, are such peptidases that mostly operate in a range of neutral to basic pH having either a metallo-type or serine-type center and are studied extensively due to their widespread utilization in detergent, food, leather and pharmaceutical applications [23]. Bacteria secreting alkaline proteases are widespread in natural [24,25,26] as well as in human-made environments [27]. However, genus *Bacillus* is one of the most indispensable group and has been extensively exploited for the alkaline protease enzyme production mainly on account of their easy isolation from varied habitats (alkalescent waters, deep sea, and soil), the potentiality to flourish on proteinaceous substrates [28], a rapid rate of growth, chemo-organotrophic growth characteristics, extracellular enzyme secretions, and safe handling [29].

Thus, the present study is a pioneer attempt intended to isolate and identify a potent protease enzyme-producing bacterial strain from the soils of lower DNP followed by extraction, purification, characterization and functional analysis of its extracellular protease through protein–protein docking to assess its properties for commercial exploitation and environmental waste management. To the best of our knowledge, there is no documentation regarding the production, characterization and substrate-binding protein–protein docking study of alkaline protease by this *Bacillus* sp. from this part of high-altitude Himalaya.

## 2. Materials and Methods

**Figure 1:**
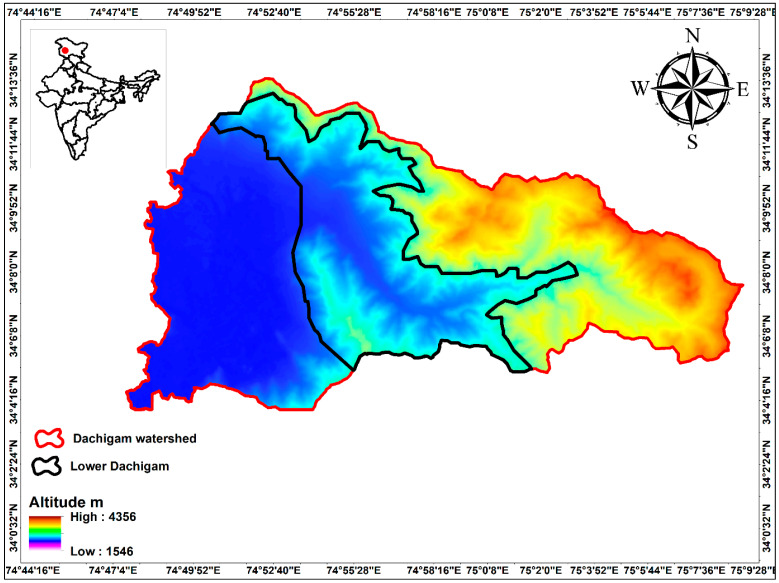
Map showing lower Dachigam National Park.

Study Site: DNP located in the North-Western Himalayan range is lying between 34°04′ N–34°11′ N and 74°54′ E–75°09′ E and stretches across an altitude of some 1677–4270 m (Figure 1). Officially the national park is sprawling over a roughly rectangular area of 141 km^2^ [30]. DNP has two ranges: lower (26 km^2^) and upper Dachigam (115 km^2^), preferably divided on the basis of the forest types, altitudinal range, and movements of Hangul deer. The present study was conducted in the lower region of DNP (also known as Lower Dachigam), comprising about one-third of its western-most portion, and consisting of a deep gorge cut across by the river Dagwan (and its tributaries) that originates in the alpine Marsar Lake (c. 4200 m) situated in Upper Dachigam (also known as the Dagwan Valley).

### 2.1. Sample Collection, Isolation and Preservation of Bacteria

Soil samples were brought seasonally for two years from selected sampling sites in lower DNP with a soil corer up to a depth of 15 cm [31], and standard serial dilution spread-plate technique was used for the isolation of culturable bacteria. The pure colonies of isolates were retrieved via subculturing and preserved into slants composed of nutrient-agar, which were then kept at 4 °C for future use [32].

### 2.2. Preliminary Screening of Isolated Bacterial Strains for the Protease Activity

All isolated bacterial strains were initially screened (in triplicates) for proteolytic activity onto the skim-milk agar plates [33]. Strain HM48, was selected for further study, as it depicted the highest hydrolytic zone of clearance around its colonies.

### 2.3. Qualitative Screening for Protease Secretion on Different Media

The qualitative enzyme screening of strain HM48 was carried out at pH 7.0 and 8.0 on different basal media (in triplicates) containing 1.0% (*w/v*) protein source of each casein, gelatin and skim milk [34]. After the incubation period, plates were analyzed for their hydrolytic zones around the bacterial colonies.

### 2.4. Identification of Strain, HM48

The strain, HM48 was identified using macromorphological [35], Gram staining, biochemical [36], and molecular approaches. This strain was also screened for the production of other hydrolytic enzymes (lipase and amylase) on respective media [37]. Antibiotic susceptibility tests using concentrations as per Clinical and Laboratory Standards Institute (CLSI) [38] were also performed for strain, HM48.

For molecular identification, the retrieved sequence of 16S ribosomal RNA from SciGenom, Labs, (Kerala) obtained after purification and sequencing of polymerase chain reaction (PCR) amplicon using universal bacterial primers [39] was blasted (BLASTn) determining its phylogenetic neighbors from the nucleotide database of National Center for Biotechnology Information, NCBI [40]. The evolutionary phylogenetic tree was made through the neighbor-joining method using maximum composite likelihood as a correction factor having a bootstrap value of 1000 replicates [41], and alignment was done by Clustal-W using MEGA 7.

### 2.5. Enzyme Production

#### 2.5.1. Media for Protease Production and Culture Conditions

Inoculation of the prepared basal media (50 mL) was done using 5% of 24 h bacterial broth culture having OD_550_ ≈ 0.2 and incubated in an orbital shaker at the rate of 120 rpm for 24 h at 37 ± 2 °C [34].

#### 2.5.2. Extraction of the Crude Proteases

After incubation, the inoculated basal media was centrifuged at 10,000 rpm for 15 min, and after discarding the cell pellet, the supernatant containing crude proteases was preserved at 4 °C and used for supplemental study [26].

#### 2.5.3. Protein Estimation and Protease Activity Assay

The concentration of protein (mg/mL) was estimated following Lowry et al. (1951) and determined as a measure of its absorbance at 700 nm after each step of the enzyme purification process, using bovine serum albumin (BSA) as a reference [42]. Protease activity of the enzyme (U/mL) was assessed as per standard protocol by Cupp-Enyard, [43] with modifications, as pH and temperature of the assay were optimized wherein the enzyme was incubated at different pH (6–12) with temperature ranging 10–90 °C. Thus, at each purification process as well as for characterization of enzyme, the protease activity was measured at a standardized pH, 8 and temperature, 70 °C during this study.

The activity was assessed by incubating 5 mL of casein (0.65% *w/v*) prepared in 0.1 M Tris-HCl buffer (pH 8.0) with 1 mL of the enzyme at 70 °C for 30 min. This reaction was stopped by adding 5 mL of 10% (*w/v*) trichloroacetic acid (TCA) and incubated for 10 min at 37 °C followed by 15 min centrifugation at 10,000 rpm (4 °C). To 1 mL of supernatant collected in a fresh tube, 2.5 mL sodium carbonate (0.4 M) and 0.5 mL Folin–Ciocâlteu reagent (1 N) were added, gently mixed and incubated at 37 °C for 30 min. The absorbance was measured at 660 nm. For calculations, one-unit enzyme activity was interpreted as the amount of enzyme required to hydrolyze the casein to release 1 μmol tyrosine mL^−1^ min^−1^ under the standard conditions of the assay. A standard tyrosine curve was used to determine the number of tyrosine equivalents released. The specific activity of the sample was calculated by dividing the enzyme units (U mL^−1^) by the overall protein content (mg mL^−1^). Thus, the sample activity was denoted in units (U mL^−1^) while the specific activity was designated as enzyme activity per mg of protein and expressed as (U mg^−1^).

### 2.6. Enzyme Purification

#### 2.6.1. Ammonium Sulfate Precipitation of Crude Proteases

The crude protease was precipitated from the supernatant using solid ammonium sulfate (40–80%) as per standard saturation levels [44]. The precipitated protein was resuspended in a small amount of 30 mM buffer (Tris-HCl, pH 7) to obtain a concentrated enzyme solution. This suspension was dialyzed with the same buffer for 12–18 h at 4 °C to remove ammonium sulfate [45]. The dialyzed sample fraction is termed as partially purified protease enzyme (PPPE).

#### 2.6.2. Gel Filtration Column Chromatography and Gel Electrophoresis (SDS–PAGE)

The PPPE extract was then purified by passing through a Tris-buffer (pH 7)equilibrated Sephadex G-50 column (Sigma-Aldrich). The PPPE was eluted using the same buffer, and 3 mL fractions (about 25 in number) at 1 mL min^−1^ flow rate were collected, and their absorbance was read at 280 nm. Those fractions that exhibited maximum protease activity were pooled, concentrated, and kept for subsequent analysis.

Gel electrophoretic analysis was executed according to Laemmli [46], and the approximate molecular weight of the protease from strain HM48 was evaluated against a standard protein ladder (Spectra, Thermo Scientific).

### 2.7. Enzyme Characterization

#### 2.7.1. Effect of Temperature and pH on Activity/Stability of Protease from Strain, HM48

The effect of temperature on enzyme activity was analyzed in a varied temperature range (10–90 °C). The thermal stability, on the other hand, was checked by pre-incubating the enzyme for 30 and 60 min at different temperatures (50, 55, 60, 65 and 70 °C) followed by measuring their percent residual activities (i.e., activity of sample after incubation/activity of sample before incubation ×100).

pH effects on the enzymatic activity were assessed using 0.1 M buffer systems comprising of phosphate buffer (pH 6–7), Tris-HCl buffer (pH 8–9) and glycine–NaOH buffer (pH 10–12), whereas the pH stability analysis was conducted by prior incubation of protease enzyme for 1 h at 35 ± 2 °C in pH buffers (6–12), and residual activities were analyzed.

#### 2.7.2. Influence of Different Substrates, Metal Ions and EDTA on Enzyme Activity of Strain, HM48

The impact of various substrates on the activity of the enzyme was investigated employing substrates (1 mg/mL), e.g., BSA, casein, gelatin and skim milk. The reaction mixtures were kept for 30 min at 70 °C, after which activity was evaluated.

The effects of several metal ions (CdCl_2_, CuSO_4_, FeCl_3_, HgCl_2_, MgCl_2_, and MnCl_2_) on the enzyme catalytic behavior was examined by its prior incubation with a specified metal ion at 1, 5 and 10 mM. Furthermore, the metal ion with which the enzyme showed maximum increase in its activity at 10 mM final concentration was examined further with 1, 5 and 10 mM of ethylene-diamine tetra acetic acid, EDTA (a chelating agent) so as to detect its impact on enzyme catalytic efficiency.

#### 2.7.3. Impact of Various Surfactants and Oxidizing Agent on Enzyme Efficiency of Strain, HM48

The effect of 0.5 and 1.0% concentrations of surfactants (sodium dodecyl sulfate, SDS in *w*/*v*; triton X-100 and tween-80 in *v*/*v*) and an oxidizing agent (hydrogen peroxide, H_2_O_2_ in *v*/*v*) on the enzyme activity was assessed, whereby reactions were kept for incubation at 35 ± 2 °C for 30 min using casein as a substrate and the residual activities were measured.

#### 2.7.4. Effect of Different Organic Solvents on Activity/Stability of Protease from Strain, HM48

The influence of organic solvents upon catalytic activity of the enzyme at 70 °C was determined using an organic solvent, namely benzene, toluene and xylene. However, their influence on protease stability was measured by pre-incubating each of these solvents with the enzyme at 35 ± 2 °C for 30 min. After this, the residual activity of the enzyme was determined at 70 °C following the optimized assay.

#### 2.7.5. Effect of Varied Casein Concentrations (Enzyme Kinetics) on Enzymatic Activity of Strain, HM48

Enzyme activity was analyzed using varying casein concentrations (0.12–2.25 mg/mL) at its optimum reaction conditions (pH, 8 and temperature, 70 °C), after which the enzyme kinetic parameter values (K_M_ and V_max_) were computed employing a Lineweaver–Burk double reciprocal plot [47].

### 2.8. Protease Gene Amplification of Strain, HM48

Extracted template DNA of strain, HM48 was used for the amplification process using a primer set [34] synthesized by Sigma-Aldrich. The standardized cycling parameters (for 50 μL reaction) included 5 min initial denaturation at 94 °C followed by 35 PCR cycles, each comprising of 30 s denaturation at 94 °C, 30 s annealing at 52 °C, 60 s extension at 72 °C and 10 min final extension at 72 °C with a post-hold at 4 °C. The electrophoresed amplicon was sent to SciGenom Labs for purification and Sanger sequencing. Protein blast (BLASTp) was utilized for identifying the retrieved sequence after determining the phylogenetic relations from the nucleotide databases in NCBI [40]. The neighbor-joining approach was used to develop an evolutionary phylogenetic relationship [41], and alignment was done by Clustal-W using MEGA 7.

#### 2.8.1. Model Generation

The structure from the amino acid sequence of strain, HM48 subtilisin-like serine protease, was generated using GalaxyTBM, of GalaxyWeb, in two stages. First, the extra reliable core structure was built through a template-based approach, following which unreliable local regions (URLs) were identified and subsequently remodeled through the thermodynamic hypothesis-based ab initio method [48]. The docking of the modeled structure was accomplished with the substrate, casein, with which this enzyme displayed maximum activity to carry out its active site analysis. For this purpose, a sequence of bovine beta (β) casein *(Bos taurus)* retrieved from UniProt (KB-P02666) was subjected to GalaxyTBM.

#### 2.8.2. Improvement of Model Quality

The best model of strain, HM48 subtilisin-like serine protease, was further refined using a highly reliable refinement software, namely GalaxyRefine, which uses a sequential approach involving side-chain rebuilding and repacking. Besides the defined functions, GalaxyRefine performs structural relaxation through its molecular dynamic simulations [49]. The GalaxyRefine models show improvement both in local as well as in global structure quality, which is seen, especially when the models to be refined have been generated through modeling software’s based on futuristic or advanced algorithms [49,50].

#### 2.8.3. Model Validation through Different Approaches

As a thumb rule, the models need to be certified prior to downstream studies to restrain errors due to defective modeling. The protease model of strain, HM48 subtilisin-like serine, was tested by a variety of methods. Its stereochemical quality was tested by PROCHECK, and the compatibility of the model (3D) to its primary structure (1D) was checked by Verify3D [51,52]. Further validation was performed by protein structure analysis (ProSA) web, an easy interface for the ProSA program [53]. Validation of bovine β-casein was also done through PROCHECK and ProSA-web.

#### 2.8.4. Protein–Protein Docking and Interaction Profile Generation

A web-based protein–protein docking server HawkDock was used for docking β-casein to strain, HM48 subtilisin-like serine protease. Molecular mechanics/generalized Born surface area (MM/GBSA) was employed to re-rank the top 10 models because of its effective re-ranking ability [54,55,56,57]. From this docked complex, the interaction profile was generated using a multifunctional web server PDBsum [58].

### 2.9. Enzyme Application

#### 2.9.1. Evaluation of Enzyme as a Detergent Additive: Enzyme Compatibility with Commercial Detergents

The detergent compatibility test with some commercially available detergents like Ghadi (RSPL Ltd., India), Surf Excel and Wheel (Hindustan Unilever Ltd., India) and Tide (Procter and Gamble Ltd., India) was carried out by incubating each of the detergent solutions (7 mg mL^−1^) with the enzyme in 1:2 ratio at the test temperature of 70 °C. The endogenic protease in the tested detergents was deactivated at 80 °C for about an hour prior to their use, after which the residual enzyme activity was estimated.

#### 2.9.2. Wash Performance Analysis: Blood and Egg Yolk Stain Removal

The wash performance of the enzyme from HM48 for stain removal on white cotton cloth pieces (5 × 5 cm^2^) was studied using detergent solutions (7 mg mL^−1^). The blood and egg yolk stained cloth (SC) pieces were separately dried in a hot oven for about 5 min (95–100 °C), and two sets (in triplicates) of below treatments each for human blood and egg yolk stained cloth pieces were prepared:

(i) 20 mL Surf Excel solution + SC piece
(ii) 20 mL Tide solution + SC piece
(iii) 20 mL Surf Excel solution + 400 μL of enzyme + SC piece
(iv) 20 mL Tide solution + 400 μL of enzyme + SC piece
(v) 20 mL tap water (T.W.) + 400 μL of enzyme + SC piece
(vi) 20 mL tap water (T.W.) + each of blood and egg yolk SC piece as control, respectively

The treatment plates were incubated at 70 °C for 30 min and at regular intervals visually checked for the removal of stains. After the incubation period, the stained cloth pieces were thoroughly rinsed, air-dried and examined for blood and egg yolk stains. The untreated blood and egg yolk stained cloth was taken as control [26].

#### 2.9.3. Application of Purified Enzyme in Waste Management: Degradation of Chicken Feather

The whole chicken feathers procured from a chicken farm were washed properly with tap water to remove blood, after which they were carefully rinsed with distilled water and autoclaved. The feathers were allowed to air-dry overnight, following which the disintegration test of the whole chicken feather was executed by incubating it with the purified enzyme at a standardized test temperature of 70 °C for about 4 h [59].

## 3. Results

### 3.1. Isolation and Preliminary Screening of Bacterial Strains for the Protease Activity

**Figure 2:**
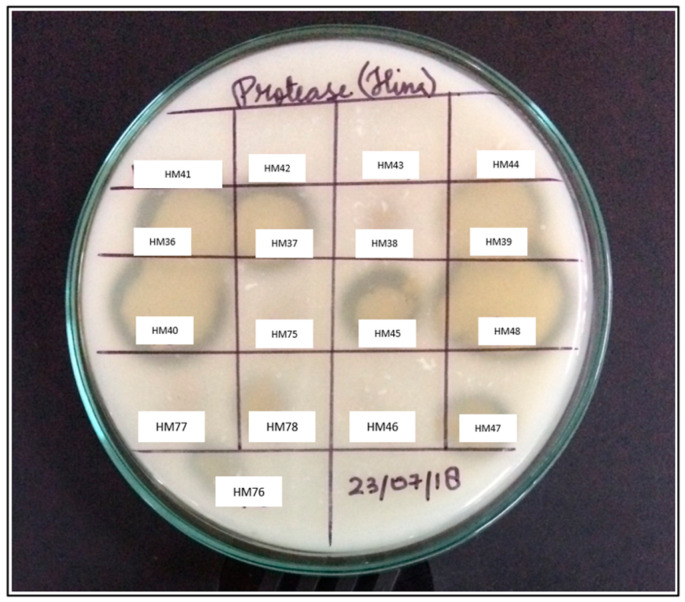
Representative image showing a preliminary screening of some isolated bacterial strains for protease on skim-milk agar.

Out of 92 isolated morphologically different strains of bacteria, 39 bacteria showed the proteolytic activity upon preliminary screening, of which isolate HM48 formed a remarkable hydrolytic zone of clearance around its colonies and thus was chosen for further study (Figure 2).

### 3.2. Qualitative Screening and Identification of Strain, HM48

#### 3.2.1. Qualitative Screening on Different Media

**Figure 3:**
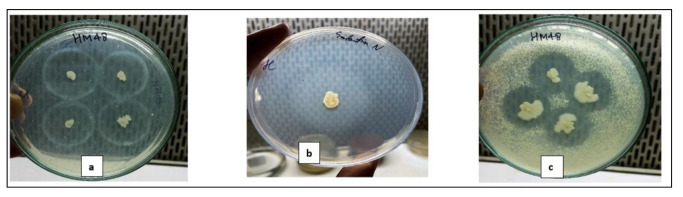
Qualitative screening of bacterial strain, HM48 for protease production using different substrates (1% *w*/*v*). Hydrolytic clearance zone on (**a**) casein; (**b**) gelatin, and (**c**) skim-milk agar plates.

The qualitative screening of isolate HM48 at pH 7.0 and 8.0 on different media revealed that it formed prominent hydrolytic zones around its colonies (Figure 3) at pH 7.0 on media containing casein (1%) as a protein source (Table 1).

#### 3.2.2. Morphological Identification

The macromorphological colony and Gram staining characteristics of strain HM48 are given in Table 2.

#### 3.2.3. Biochemical Identification

The biochemical identification results revealed that isolate, HM48 was able to utilize monosaccharides such as D and L-arabinose, fructose, galactose, mannose, rhamnose, sorbose, and xylose except for dextrose. Moreover, it was capable of utilizing monosaccharide derivative, esculin, but malonate and salicin were not utilized by it. This strain was also found to be inefficient in utilizing several di- and tri-saccharides except for cellobiose, citrate, inositol, inulin, mannitol, sodium gluconate, sorbitol, sucrose, and trehalose. The antibiotic susceptibility tests revealed that this strain was extremely sensitive to all the tested antibiotics (Table 3). In addition, isolate HM48 was able to produce hydrolytic enzymes, lipase and amylase as depicted by clear zones around its colonies.

#### 3.2.4. Molecular Identification

**Figure 4:**
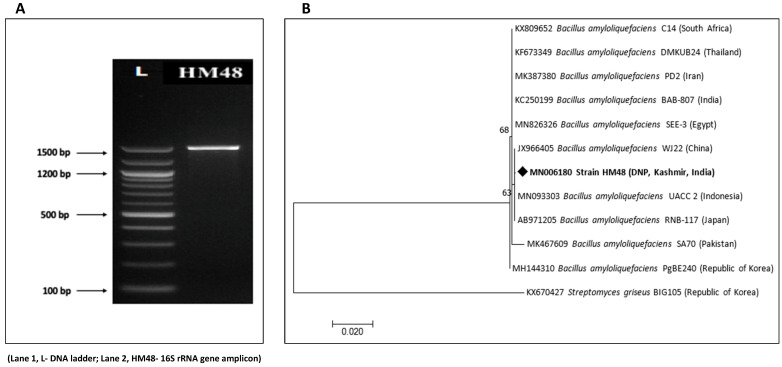
Molecular identification of *B. amyloliquefaciens*, HM48 based on its 16S rRNA gene sequence. (**A**) Representative electrophoretic image of amplicon; (**B**) neighbor-joining phylogenetic tree.

The unidirectional 16S rRNA gene analysis, a highly conserved gene (A) which is widely used for prokaryotic species identification, retrieved a nucleotide sequence of 885 bp that was submitted to GenBank having accession number MN006180. The sequence was checked on the NCBI database using nucleotide BLASTn depicting 100% similarity with the *Bacillus amyloliquefaciens* species, and its evolutionary proximity with them is clearly visible in Figure 4B.

### 3.3. Purification of Enzyme and Molecular Weight Determination of Protease from Strain, HM48

**Figure 5:**
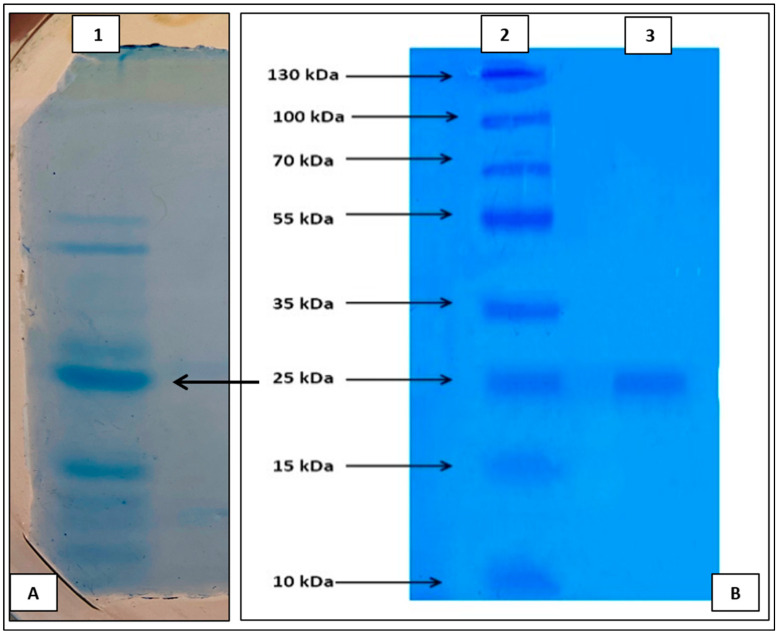
Representative image of SDS–PAGE of (**A**) crude and (**B**) purified protease enzyme from *B. amyloliquefaciens*, HM48 (lane 1—crude enzyme; lane 2—protein marker; lane 3—purified enzyme).

The supernatant was utilized as a crude enzyme source, which was then precipitated using 40–80% standard ammonium sulfate saturation levels. However, 80% saturation turned out to be the best percentage for precipitating the crude proteases. This precipitated enzyme was subjected to subsequent purification via dialysis and gel filtration chromatography. The specific activity of the culture supernatant (10.54 U mg^−1^) was enhanced to 22.81 U mg^−1^ and 38.54 U mg^−1^ by ammonium sulfate precipitation and dialysis, respectively, after which the column chromatographic purification raised it to 61.05 U mg^−1^ resulting in a purification fold of 5.8 with a yield of about 36.6% (Table 4). As per the SDS–PAGE, the estimated molecular weight of enzyme from strain, HM48, was around ≈25 kDa as indicated by the manifestation of one band in the gel (Figure 5).

### 3.4. Enzyme Characterization

#### 3.4.1. Effect of Temperature on Activity/Stability of Protease from Strain, HM48

**Figure 6:**
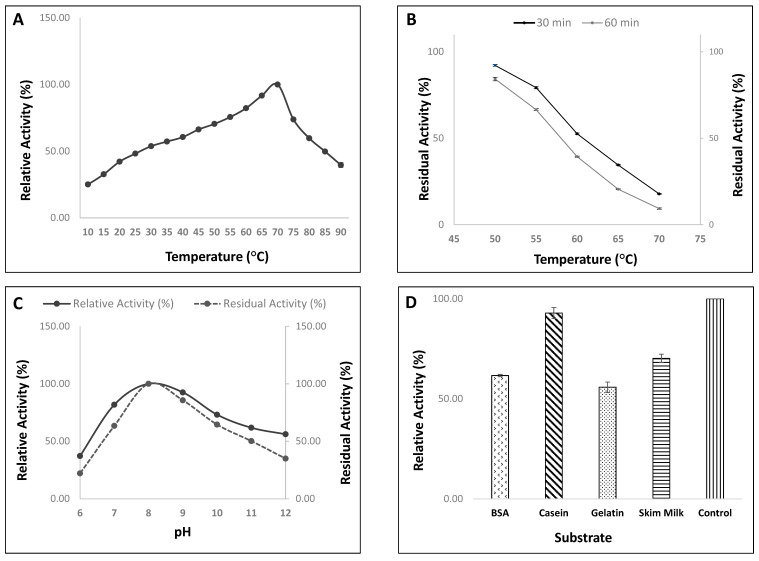
Evaluation of activity/stability of protease from *B. amyloliquefaciens* HM48 with varying (**A-enzyme activity)**, (**B-enzyme stability**) temperature, (**C**) pH, and (**D**) substrates.

The study on enzyme optimal temperature of strain, HM48 revealed that this enzyme was active over varied temperatures (10–90 °C), peaking at 70 °C, thereby supporting the novel heat-stable attribute of the enzyme (Figure 6A). A continuous increase was observed from 10 °C up to a temperature of 70 °C after-which any further increase in temperature decreased the activity substantially. However, at temperatures 10 °C and 90 °C, the enzyme exhibited 25.1% and 39.61% relative activity, respectively, with regard to 100% relative activity at an optimum temperature of 70 °C.

Thermal stability studies conducted in the temperature range of 50 to 70 °C (Figure 6B) reflected the stable nature of enzyme at low temperature (up to 50 °C), retaining 92.15% of its activity within 30 min and 84.25% post 60 min incubation at 50 °C. However, this enzyme swiftly lost most of its activity above 50 °C, suggesting the thermal inactivation of the enzyme by heat as the incubation time increases. Even at its optimum activity temperature of 70 °C, the enzyme maintained only 17.74 and 9.29% of its activity after 30 and 60 min, respectively.

#### 3.4.2. Effect of pH on Activity/Stability of Protease Enzyme from Strain, HM48

Ideal pH of protease was assessed using various buffers, results of which revealed the activeness of the enzyme over a wide pH range (6–12), reaching its peak at pH 8.0 with relative activity of 100%. Enzyme activity was observed to elevate proportionally from pH 6.0 to pH 8.0, after which any further deviation in the pH value reduced the activity significantly. Moreover, with the change in pH towards the acidic end (pH 6.0), a progressive decrease in the relative activity (%) was observed. The protease enzyme from strain HM48 showed an activity of 81.8%, 92.5% and 73.1% at pH 7.0, 9.0, and 10, respectively, in relation to the activity at pH 8.0. However, with a shift in pH towards alkalinity, the enzyme showed an activity of 61.8% (pH 11) and 56.1% (pH 12). On the other hand, the pH-dependent stability profile of this protease revealed its stability from pH 8–11, preserving its maximal residual activity at pH 8.0 while as considering pH 6.0 and 12, only 22.20% and 35.08% of activity (as regards to the activity at pH 8.0) was measured (Figure 6C).

#### 3.4.3. Effect of Different Substrates on Enzyme Activity of Strain, HM48

Of all tested substrates (BSA, casein, gelatin, and skim milk), the enzyme hydrolyzed almost all proteins making it a distinct feature of this novel enzyme from strain, HM48. However, the substrate with which the enzyme depicted its highest activity (92.87%) was casein (Figure 6D).

#### 3.4.4. Influence of Various Metal Ions and EDTA on Enzyme Activity of Strain, HM48

**Figure 7:**
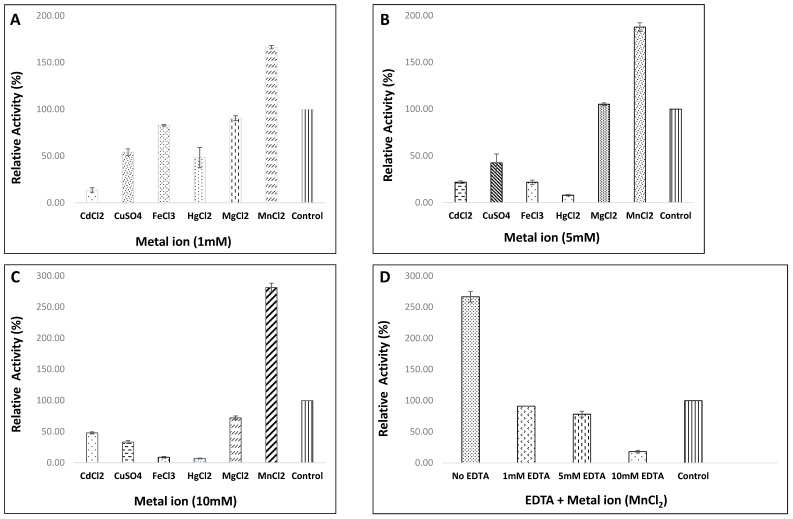
Effect of metal ions, at (**A**) 1.0, (**B**) 5.0 and (**C**) 10 mM final concentrations and (**D**) EDTA (10 mM) in the presence of 10 mM final concentration of metal ion, MnCl_2_ on the activity of protease from *B. amyloliquefaciens* HM48.

The effects of these ions tested at different concentrations are given in Figure 7A–C. Among the metal ions, Mn^2+^ at all concentrations regulated the enzyme activity positively while remaining ions such as Cd^2+^, Cu^2+^, Fe^2+^, and Hg^2+^ regulated it negatively. In contrast, the addition of 1.0, 5.0, and 10 mM final concentration of Mn^2+^ ion to the enzyme enhanced its protease activity by 66.48, 87.85 and 181.33%, respectively. The Hg^2+^ ions, on the contrary, significantly reduced it from 48.17% at 1.0 mM to 7.99% at 5.0 mM, reaching its maximum inhibition (6.99%) at 10 mM concentrations. However, Mg^2+^ was found to be inhibitory at 1- and 10 mM concentrations, while it tended to increase the enzyme activity by 5.23% at 5 mM concentration. During the present study, the inhibiting effect of EDTA was noticed, as the percent enzyme activity was 91.21% at 1.0 mM EDTA concentration, which was subsequently reduced to 78.27 and 18.19% at 5.0 and 10 mM concentrations of EDTA, respectively, thereby suggesting the requirement of metal ion (like Mn^2+^) for optimal activeness of this protease (Figure 7D).

#### 3.4.5. Effect of Various Surfactants and Oxidizing Agent on Enzyme Efficiency of Strain, HM48

**Figure 8:**
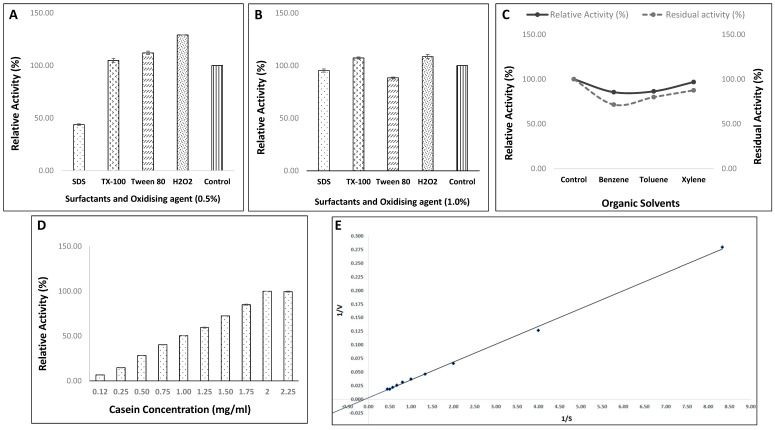
Evaluation of activity of protease from *B. amyloliquefaciens* HM48 in the presence of various surfactants and an oxidizing agent at (**A**) 0.5%, and (**B**) 1.0% final concentration; (**C**) different organic solvents; (**D**) varying casein concentration and (**E**) Lineweaver–Burk plot.

This was mainly done to investigate whether the enzyme is compatible and stable in the presence of different surfactants and oxidizing agents for assessing its applications in industries, particularly for laundry purposes. During the present investigation, apart from SDS, the alkaline protease enzyme was quite stable with almost all the assayed surfactants. At both concentrations, SDS, an anionic surfactant, inhibited the activity of this protease, retaining about 43.76 and 95.31% activity at 0.5 and 1.0% concentration, respectively. Triton-X 100 (a nonionic detergent) enhanced the activity from 4.84% at 0.5% and 7.30 at 1.0% concentrations. Moreover, Tween-80, a nonionic surfactant, was found to enhance and then inhibit the enzyme activity from lower (0.5%) to higher concentration (1%). On the other hand, the oxidizing agent, H_2_O_2,_ depicted an increase of 29.07% in enzyme activity at 0.5% concentration, which was drastically reduced to 8.53% at 1.0% concentration (Figure 8A,B).

#### 3.4.6. Effect of Various Organic Solvents on Activity/Stability of Protease from Strain, HM48

The influence of different organic solvents exhibited activity was marginally affected in the presence of xylene (96.78%), while toluene (86.4%) and benzene (85.49%) had a moderate effect on it. Furthermore, the enzyme stability studies revealed that the enzyme showed remarkable stability with each of the tested organic solvents, retaining its maximum residual activity with xylene (87.53%) followed by toluene (79.94%) and benzene (71.54%), as shown in Figure 8C.

#### 3.4.7. Effect of Varied Casein Concentrations (Enzyme Kinetics) on Activity of Protease from Strain, HM48

The enzyme activity ascertained at standardized optimum reaction conditions with varying concentrations of substrate (casein) depicted its maximum at 2 mg/mL casein concentration (Figure 8D). A swift increase in the reaction rate was noticed, particularly from 0.5 to 2.00 mg/mL, after which the rate was found to get stabilized. Moreover, the values of enzyme kinetic parameters for this alkaline proteolytic enzyme as determined by Lineweaver–Burk plot were computed to be 11.71 mg mL^−1^ (K_M_) and 357.14 µmol mL^−1^ min^−1^ (V_max_), respectively (Figure 8E).

### 3.5. Identification of Protease Gene

**Figure 9:**
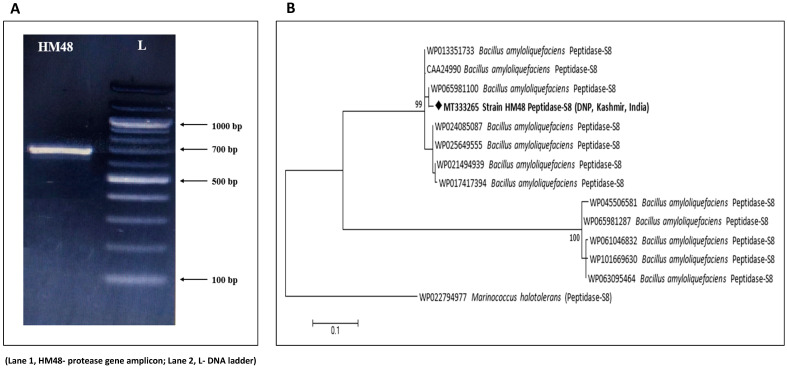
Molecular identification of *B. amyloliquefaciens* HM48 based on its protease gene sequence. (**A**) Representative electrophoretic image of amplicon; (**B**) neighbor-joining phylogenetic tree.

As shown in Figure 9A, amplification of this gene yielded a product of about 700 bp, which when checked against available sequences in NCBI via BLASTp displayed a similarity of 99.03% with subtilisin-like serine alkaline protease of *Bacillus amyloliquefaciens*. The retrieved nucleotide sequence was deposited under accession number MT333265 in GenBank, NCBI after-which this gene sequence was translated and modeled, followed by molecular docking with a specific substrate. The tree of phylogeny was constructed employing MEGA 7 (Figure 9B).

#### 3.5.1. Model Generation, Improvement and Validation

**Figure 10:**
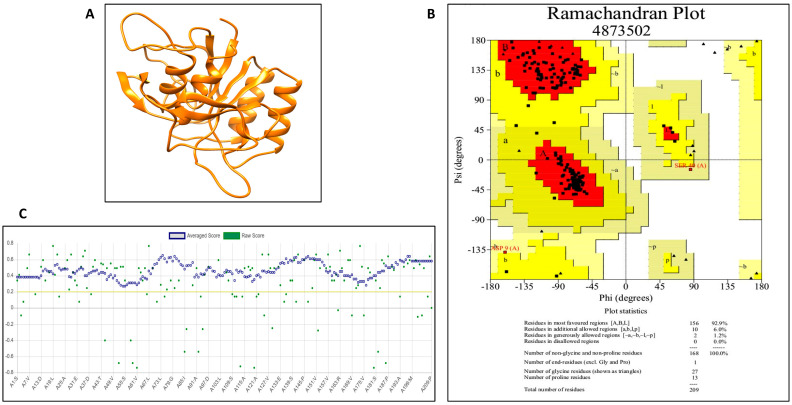
(**A**) Refined model of *B. amyloliquefaciens* HM48 subtilisin-like protease. The model was generated through GalaxyTBM, refined through GalaxyRefine and rendering was done with the help of UCSF Chimera; (**B**) Ramachandran plot of GalaxyRefine model of *B. amyloliquefaciens* HM48 subtilisin-like protease. As per the plot statistics, the model quality is quite good and is thus highly reliable for further studies; (**C**) verify 3D analysis of GalaxyRefine model of *B. amyloliquefaciens* HM48 subtilisin-like protease. One hundred percent of the residues displayed average score greater than or equal to 0.2. The rule of thumb is that a minimum of 80% of the residues should possess an averaged 3D-1D score of ≥ 0.2. This further indicates the compatibility between 3D structure and primary structure (amino acid residue sequence).

**Figure 11:**
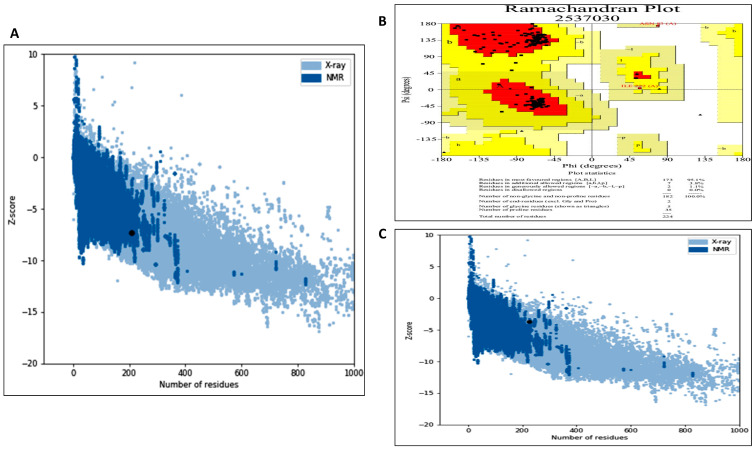
(**A**) ProSA-web analysis of the refined model of *B. amyloliquefaciens* HM48 subtilisin-like protease. Z-score of this model (−7.34) predicted by the ProSA program was found to be well within the range typically observed for proteins (native) of similar size. Z-scores lying outside the range typical for native proteins specify inaccurate structures; (**B**) Ramachandran plot of the refined model of bovine *β-casein* authenticates the high stereochemical quality of this model. For a good quality model above 90% of the residues shall fall in most favored regions, and in our case, 95.1% of the residues were found in the defined regions, clearly indicating the quality of the model to be good; (**C**) ProSA-web z-score plot of β-casein (bovine) model. Z-score is indicative of the general model quality, and if this score of the model falls within the range typically displayed by native proteins bearing similar size, the model is expected to be correct, and if the Z-score of the model violates the defined range, then such model is probably erroneous. From the plot, it is quite evident that the Z-score of the β-casein model having 224 amino acid residues is −3.67 and falls within the confines of the reference range.

The protein model of *Bacillus amyloliquefaciens* HM48 subtilisin-like serine protease was generated through GalaxyTBM, which was improved by GalaxyRefine (Figure 10A), and rendering was done using UCSF Chimera. Ramachandran plot of this modeled protein contained 92.9% residues in the most favored region, thus reflecting its stability (Figure 10B). The quality was further validated by Verify 3D results (Figure 10C) as 100% residues exhibited averaged score (3D-1D) ≥ 0.2 as well as ProSA-web with Z-score calculation of −7.34 (Figure 11B). The bovine β-casein model also passed the Ramachandran plot with 95.1% residues in the favorable region (Figure 11A) and ProSA-web analysis with −3.67 Z-score (Figure 11C).

#### 3.5.2. Protein–Protein Docking

**Figure 12:**
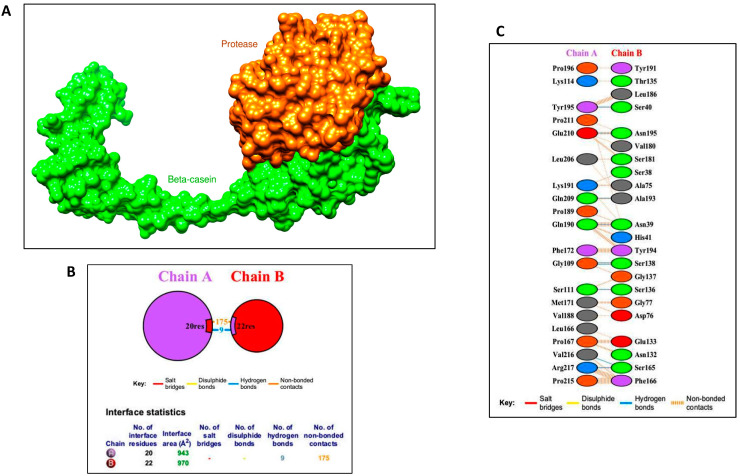
(**A**) Docked complex of β-casein and *B. amyloliquefaciens* HM48 subtilisin-like protease rendered with the help of UCSF Chimera software. Green colored region indicates β-casein; the remaining color designates the *B. amyloliquefaciens* HM48 subtilisin-like serine protease; (**B**) residues of β-casein (chain A) interacting with *B. amyloliquefaciens* HM48 subtilisin-like protease (chain B). Twenty residues of this casein interact with 22 residues of HM48 subtilisin-like protease; (**C**) detailed account of interactions between β-casein residues (chain A) and *B. amyloliquefaciens* HM48 subtilisin-like protease (chain B). Following docking with HawkDock, the interaction profile was generated with the help of PDBsum.

Among the top 10 docked complexes, the complex with the binding free energy of −48.43 (kcal/mol) seemed to be logical based on experimental evidence (Figure 12A). Though this evidence is sufficient to prove that β-casein binds to *Bacillus amyloliquefaciens* HM48 subtilisin-like serine protease at the active site, but it is still debatable whether the region of β-casein interacting with the active site of this extracellular serine protease is correct or not. Interaction profile study showed that 22 residues of *Bacillus amyloliquefaciens* HM48 subtilisin-like serine protease interact with 20 residues of β-casein. Nine hydrogen bonds were detected between the two proteins (Figure 12B,C).

### 3.6. Application of Enzyme from Strain, HM48

#### 3.6.1. Evaluation of Enzyme as a Detergent Additive: Enzyme-Detergent Compatibility and Wash Performance Analysis

**Figure 13:**
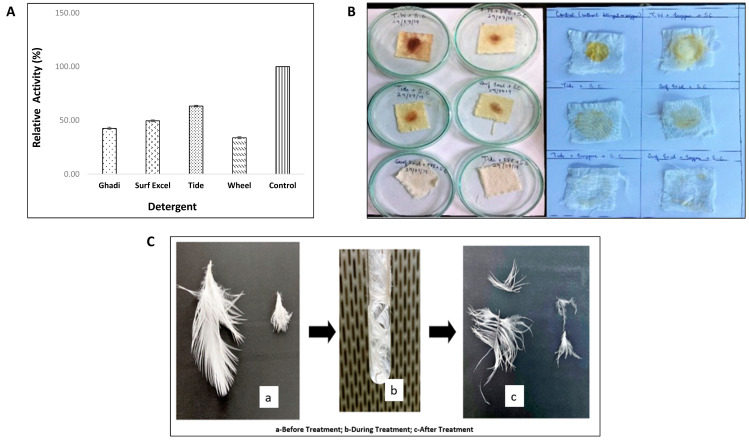
Evaluation of protease from *B. amyloliquefaciens* HM48 for industrial and environmental application. (**A**) Detergent compatibility; (**B**) wash performance analysis (blood and egg-yolk stain removal, and (**C**) waste management (chicken feather degradation).

The detergent affinity tests of the enzyme (Figure 13A) suggested that this alkaline protease was most compatible with Tide, retaining 63.28% of activity even when exposed to a temperature of 70 °C for 30 min thus clearly reflecting its potential use in the production of commercial detergents. These results were confirmed by complete blood, and egg yolk stain removal (Figure 13B) with Tide conducted as a part of this study.

#### 3.6.2. Evaluation of Protease Enzyme for Application in Waste Management: Chicken Feather Degradation

The feather disintegration study suggested that the alkaline protease enzyme from strain HM48 hydrolyzed a considerable amount of protein and thus was efficient in degrading the chicken feathers to a great extent (Figure 13C).

## 4. Discussion

In soil, the principal sources of proteases vary from microorganisms [60], plants [61], and animal excreta [62] to the decomposition of organic matter, and dry-wet deposition [63] and the chief microbial proteases (serine-alkaline proteases, subtilisin’s and subtilisin-like proteases) are mainly produced by *Bacillus* and *Pseudomonas* strains of bacteria [64]. Microbial proteases mostly have their optimum catalytic pH in the 3.0–12 range [61]; however, those isolated from soil microorganisms retain their maximum activities at optimum temperatures between 40 and 60 °C within pH 8–9 [65]. The present study was thus aimed at identifying a high pH and thermo-tolerant protease producing bacterial strain from the soils of lower Dachigam National Park, Kashmir, through various culture-based analysis and protease characterization assays to assess its bioprospecting potential. The strain, HM48, was identified as *Bacillus* sp. by colony characterization as per Bergey’s manual of systematic bacteriology [35] and the same was confirmed by 16S rRNA analysis as it showed the highest similarity (100% identity) with species databases of *Bacillus amyloliquefaciens* in NCBI. The strain, HM48 was found to be extremely sensitive to the tested antibiotics, including cephalosporin’s (cefoxitin, ceftazidime, cephalothin), penicillin’s (amoxicillin/clavulanic acid or amoxiclav, oxacillin and penicillin-G), vancomycin and teicoplanin that cause inhibition in the cell wall formation by preventing the transpeptidation reaction during peptidoglycan synthesis. It was also sensitive towards various common aminoglycoside (gentamicin) and macrolide antibiotics (clindamycin and erythromycin) that can cause the inhibition of peptide chain elongation during protein synthesis. Furthermore, this strain was found to be sensitive to fluoroquinolone antibiotics such as ofloxacin, leading to inhibition of nucleic acid synthesis by blocking the replication and transcription of DNA [66].

In enzyme studies, purification and characterization of the enzyme are of vital importance for a better understanding of their nature and efficient application in industry as well as waste processing practices. During this study, the protease enzyme was purified via several purification processes, enhancing its purification by 5.8-fold and increasing its specific activity to 61.05 Umg^−1,^ as also reported by Ahmad et al. [8]. A study by Thebti et al. [67] suggested that ideal specific activity is usually attained at elevated ammonium sulfate precipitations that go up to 90% sometimes, and a similar finding was made in this study whereby 80% of the precipitation was found to yield the optimal specific activity [68]. Interestingly, after each successive purification, a decrease in the total activity, as well as the total protein content was observed, which is consistent with other works on proteases [69]. Likewise, enzyme recovery yield was also observed to decrease with every purification step on account of either the autolysis of the enzyme or removal of certain low specific activity proteases [70]. Although processes of protease purification may vary, in most of the studies, precipitating (ammonium sulfate) and dialyzing enzyme along with size exclusion column chromatography [71] are mostly used either separately or in combination so as to enhance the purity of the enzyme [68].

Based on SDS–PAGE, the molecular weight (MW) of the purified enzyme of *B. amyloliquefaciens* HM48 was around ≈25 kDa, which was undoubtedly in compliance with the study by Sai-ut et al. [72]. Many previously reported literatures also suggest low molecular weight proteases (<30 kDa) from the genus, *Bacillus* including *B. megaterium* (MW-28 kDa, [70]), *B. cereus* S8 (MW-21.8 kDa, [73]), and *B. subtilis* APMSU6 (MW-18.3 kDa, [74]).

Studies on the temperature profile of enzymes by *B. amyloliquefaciens* HM48 revealed the flexibility of the enzyme remaining active across varied temperatures (10–90 °C). The enzyme activity enhanced continuously from 10 °C attaining its maximum at 70 °C, above which it decreased drastically, and these results were much higher than previously reported 50 °C (10,72] and 60 °C [26,75] for the protease enzyme activity from several species of *B. amyloliquefaciens*. Enzymes, when exposed to higher temperatures for a prolonged time, may undergo conformational changes leading to their denaturation [76]. It is a well-known fact that the thermostability of an enzyme is established by various factors, such as distinctive composition and sequence of amino acids, ionic interactions between them, degree of hydrogen bonds and reduction in the hydrophobic surface area [77]. Various research works have extensively assessed the means of stabilizing enzymes whereby they corroborated the significance of these salt bridges in the thermal stability of proteins [78,79], and the same has been authenticated by the studies which suggest that mesophilic proteins have less number of salt bridges in comparison to their thermophilic counterparts [80]. Enzyme thermostability is also affected by several intrinsic factors, including molecular interactions concerning hydrophobic, hydrogen, ionic and metal binding, as well as extrinsic conditions, including environmental factors such as activators, cofactors, substrate, protein concentration, and also the presence of specific salts [81]. Thermal stability studies of *B. amyloliquefaciens* HM48 reflected that although the enzyme maintained 92.15% and 82.45% of activity post 30 and 60 min incubation (50 °C), respectively, it constantly decreased up to its optimum of 70 °C, which could be due to the self-hydrolyzing of amino acids in the protein chains. Therefore, it is evident that the optimum activity temperature of *B. amyloliquefaciens* HM48 was beyond many previously recorded *Bacillus* derived proteases [82,83], suggesting that this alkaline protease could be exploited for high-temperature (up to 70 °C) industrial applications.

Proteases, based on active pH optimum, are grouped into acidic, neutral, and basic (or alkaline) proteases [84]. Of all these, alkaline proteases are particularly important as they remain active in neutral to alkaline pH range and are widely exploited in cosmetic, detergent and leather industries [23]. However, their exploitation in these industries is mostly limited due to their inability to withstand elevated pH and temperature, along with detergent additives and several organic solvents [85]. Thus, exploration of novel bacteria producing highly active, heat-stable alkaline protease has gained much research attention [8,86]. During the present study, the enzyme from *B. amyloliquefaciens* HM48 was stable with pH buffers ranging from acidic to alkaline (pH 6–12), thereby reflecting that the enzyme has a wide pH spectrum reaching its maximal activity at pH 8.0, and an analogous observation has been recorded by Guleria et al. [75]. However, the enzyme under examination had a much wider alkaline pH range than many previously reported *B. amyloliquefaciens* species having pH in the range of 5.0–11 [27], 4.0–10 [10], 4.0–10 [72], 6.0–10 [83] and 6.0–11 [26]. On the other hand, the pH-dependent enzyme stability profile of *B. amyloliquefaciens* HM48 (pH 8–11) also did not display much variation in the residual activity of the enzyme, thereby suggesting stability of the enzyme in alkaline pH range. Therefore, proteases from strain HM48, like some other reported microbial proteases, were found to have profound activity mainly in the alkaline range [87] that could pave the way for its exploitation in industries.

Casein is a commonly utilized substrate in protease studies [88] as it is subjected to proteolysis with almost all proteolytic enzymes without any need of denaturing initially mainly due to its intricate arrangement and arbitrary structure [89]. During the study, substrate, casein showed the highest enzyme activity among different tested substrates, which are in accordance with findings of Mothe and Sultanpuram [83]. Several types of research also emphasized casein, being the ideal substrate [82] as milk proteins like casein, is a great source of amino acids for the growth of high cell masses [90]. Alkaline proteases require several metal ions to retain their stable nature at a higher temperature, which generally functions as a cofactor by acting as an ion or salt bridge between two amino acids, thereby preserving the exact conformation (active forms) of enzyme molecules [91]. Among the tested metal ions, only Mn^2+^ ion positively regulated the activity of the protease enzyme from strain HM48 at all concentrations [10], as manganese ions are reported to stabilize the molecular structure of enzymes in some *Bacillus cereus* derived proteases [92]. However, Hg^2+^ markedly reduced the enzyme activity from lower (1 mM) to highest (10 mM) concentrations, either by binding of Hg^2+^ to the critical side-chains of protein changing its active site structure or by causing general unfolding [93] and a similar trend has been advocated by Ibrahim et al. [94]. Various researchers have reported the positive as well as negative effects of metal ions like Cd^2+^, Cu^2+^, Fe^2+^, and Hg^2+^, Mg^2+^ and Mn^2+^ ions on enzyme activities from several *Bacillus* species [86,95]. EDTA, a chelating agent, is a water softener widely utilized as a detergent additive for removing various stains. A study on the inhibitory action of EDTA on the enzymatic activity of *B. amyloliquefaciens* HM48 concluded that it lost most of its activity when treated with different concentrations of EDTA, reaching its lowest with 10 mM EDTA concentration, thus suggesting that the enzyme could belong to serine alkaline proteases and our findings were in compliance with EL-Eskafy et al. [96].

High-quality detergent stable proteases must be congenial with most of the detergent additives, including bleach, oxidizing agent, surfactant, etc. [17]. Although anionic surfactant, SDS has been reported to have a negative influence on the protease enzyme activity [75,94], enzymes under the current study retained > 95% of the activity with SDS (1%). Both of the nonionic surfactants (triton-X100 and tween-80), however, were found to increase the activity from 4–12%, respectively. Mainly, proteases belonging to the genus *Bacillus* are generally unstable in the presence of oxidizing agents like H_2_O_2_ [97], yet the enzyme from *B. amyloliquefaciens* HM48 depicted an increase of 8–29% in its activity against H_2_O_2_. Thus, one of the promising aspects of this protease from *B. amyloliquefaciens* HM48 was its affinity with tested surfactants and oxidizing agents, as only a few wild microorganisms having compatibility, particularly with SDS and oxidizing agents, H_2_O_2_ are known [68,98]. Interestingly, the enzyme under investigation exhibited remarkable activity and stability, particularly towards xylene, retaining about > 96% relative activity and > 87% of residual activity, as organic solvents are known to stabilize the protease enzymes mainly by replacing few water molecules with organic ones resulting in stabilization of its conformation [99].

The enzyme kinetic attributes (K_M_ and V_max_) of *B. amyloliquefaciens* HM48 were ascertained adopting Lineweaver–Burk double reciprocal plot. These values mostly rely on the enzyme source (microorganism) and the type of substrate, thereby indicating the sensitive nature of the enzyme against its substrate, which is believed to increase as the K_M_ value increases while the V_max_ value decreases [100]. Values of K_M_ (11.71 mg mL^−1^) and V_max_ (357.14 µmol mL^−1^ min^−1^) were computed with good correlation (R2 = 0.9986), which was higher than many other previously reported values of K_M_ and V_max_ for several bacterial species as 3.846 mg mL^−1^ and 76.923 U mL^−1^ min^−1^ [101]; 3.99 mg mL^−1^ and 41.49 U mL^−1^ [102].

As per MEROPS database on peptidases (http://merops.sanger.ac.uk/), members of subtilisin-like proteases (subtilases) broadly categorized as S8 sub-family of the serine proteases super-family, possess immensely preserved arrangement of amino acids in the active site whereby neighboring as well as oppositely charged pair residues interact with each other forming hydrogen bonds or salt bridges, that affect a variety of their structural and functional traits. The model of *B. amyloliquefaciens* HM48 subtilisin-like serine protease showed sound stereo-chemical quality as authenticated by the Ramachandran plot with 92.9% residues in the most favored region while only 6.0% residues occurred in the additional allowed region [103]. The high quality of this model was also certified by Verify 3D results in which 100% residues exhibited averaged score (3D-1D) ≥ 0.2, the minimum criteria being only 80% [104], thus indicating the compatibility between 3D structure and primary structure (amino-acid sequence). Evaluation of *B. amyloliquefaciens* HM48 subtilisin-like serine protease refined model through ProSA-web (Z-score calculation) further supported its correctness as the Z-score observed was well within the range of native proteins possessing similar size [105]. The bovine β-casein model also passed the stereochemical quality test successfully through the Ramachandran plot and ProSA-web, thus validating its accuracy [53].

The compatibility test with locally available commercial detergents suggested that the enzyme was most compatible with Tide detergent, retaining 63.28% of its activity, and the same was validated by the wash performance analysis of the enzyme. Furthermore, from the results of chicken feather disintegration, it could be inferred that this enzyme can be exploited for converting waste chicken feathers into protein hydrolysate, thereby helping in proper chicken feather waste management from local poultry houses. Moreover, this protein hydrolysate may contain beneficial proteins that can be used in the production of organic fertilizers and animal fodder [59,106].

## 5. Conclusions

This study reports isolation, selection, characterization and functional analysis of novel alkaline protease enzyme-producing *B. amyloliquefaciens* HM48 from soils of lower Dachigam National Park, Kashmir Himalaya. The purification and characterization studies of the extracted protease exhibited its active and stable nature over a wide range of pH and temperature with an optimum at 8.0 and 70 °C, respectively, even in the presence of several metal ions, inhibitors, surfactants, oxidizing agent and organic solvents. This stable temperature enzyme displayed compatibility with almost all of the tested commercial detergents after pre-incubating it with them at a high-temperature (70 °C). Such characteristics advocate it as a potential alternative, especially in industrial applications requiring elevated temperatures for desired results. The improved wash performance displayed by the complete removal of blood and egg stains with the enzyme-detergent formulation reflects that this enzyme may find its application as a detergent additive. On the other hand, a disintegration study of chicken feathers suggested successful exploitation of this novel heat-stable protease for environmentally sustainable and efficient management of waste chicken feathers, the protein hydrolysate of which could be used for largescale production of organic fertilizers and animal fodder. Lastly, the protein–protein modeling of *B. amyloliquefaciens* HM48 helped us in understanding its catalytic behavior whereby the results revealed its subtilisin-like serine nature. The protein–protein docking analysis of this enzyme with its ideal substrate, casein, reflected its active site, which could pave the way for a better understanding of its nature for different prospective industrial and environmental exploitations.

## Figures and Tables

**Table 1:** Qualitative screening of strain HM48 for its proteolytic activity on different proteinaceous substrates (pH 7.0).

Proteinaceous Substrate	Mean Diameter of Colony, d (in mm)	Mean Diameter of Hydrolytic Zone, D (in mm)	D/d (in mm)
Casein	3	28	9.33
Gelatin	10	33	3.3
Skim milk	7	26	3.71

**Table 2:** Morphological characteristics of strain, HM48.

Size	Shape	Margin	Elevation	Texture	Appearance	Color	Transparency	Gram’s Reaction	Cell Shape	Cell Arrangement
Moderate	Irregular	Undulate	Umbonate	Rough	Dull	Cream	Opaque	Positive	Bacilli	Streptobacilli

**Table 3:** Carbohydrate utilization and antibiotic susceptibility tests of strain, HM48.

Carbohydrate	Result	Antibiotic	Concentration *	Result
Dextrose utilization	Positive	Amoxiclav (amoxicillin/clavulanic acid) (AMC)	30 mcg (20/10 mcg)	Sensitive
Esculin hydrolysis	Positive	Cefoxitin (Cephoxitin) (CX)	30 mcg	Sensitive
Cellobiose utilization	Positive	Ceftazidime (CAZ)	30 mcg	Sensitive
Citrate utilization	Positive	Cephalothin (CEP)	30 mcg	Sensitive
Inositol utilization	Positive	Clindamycin (CD)	2 mcg	Sensitive
Inulin utilization	Positive	Erythromycin (E)	15 mcg	Sensitive
Mannitol utilization	Positive	Gentamicin (GEN)	10 mcg	Sensitive
Sodium gluconate utilization	Positive	Ofloxacin (OF)	5 mcg	Sensitive
Sorbitol utilization	Positive	Oxacillin (OX)	1 mcg	Sensitive
Sucrose utilization	Positive	Penicillin-G (P)	10 units	Sensitive
Trehalose utilization	Positive	Teicoplanin (TEI)	30 mcg	Sensitive
		Vancomycin (VA)	30 mcg	Sensitive

* Concentration of antibiotics as per Clinical and Laboratory Standards (CLSI, https://clsi.org/).

**Table 4:** Purification process of enzyme produced by *B. amyloliquefaciens* HM48.

Purification Process	Total Activity (U mL^−1^)	Total Protein (mg mL^−1^)	Specific Activity (U mg^−1^)	Yield (%)	Purification Fold
Culture supernatant	313.18	29.70	10.54	100	1
Ammonium sulfate precipitation	215.63	9.45	22.81	68.9	2.2
Dialysis	160.06	4.15	38.54	51.1	3.7
Gel filtration chromatography	114.49	1.88	61.05	36.6	5.8

## Data Availability

The data presented in this study are available on request from the corresponding author.
